# Investigating the Impact of the Secretome From hAMSCs on HT‐29 Colon Cancer Cells via TNF‐α/TGF‐β/c‐MYC Signaling Pathways Using a Three‐Dimensional Cell Culture Model

**DOI:** 10.1002/iid3.70283

**Published:** 2025-10-15

**Authors:** Mohammad Ali Majdoddin, Fatemeh Safari

**Affiliations:** ^1^ Department of Biology, Faculty of Science University of Guilan Rasht Iran

**Keywords:** 3D cell culture model, hAMSCs, HT‐29 cancer cells, IL‐4/6/8 expression, TNF‐α/TGF‐βR/c‐MYC signaling pathways

## Abstract

**Background:**

Colon cancer is recognized as one of the predominant reasons of death globally. The ongoing cancer treatment techniques have not been successful; so, a strong and unique platform is needed. Interestingly, it has been discovered that stem cells offer a valuable and promising platform in cancer treatment. The goal of this study is to explore the influences of hAMSCs secretome on HT‐29 colon cancer cells by analyzing TNF‐α/TGF‐βR/c‐MYC signaling pathway, expression of interleukins (IL)‐4/6/8, and cyclin‐dependent kinase (CDK) inhibitory proteins (p15^INK4B^ and p21^CIP1^) using a hanging drop 3D cell culture model.

**Methods:**

In this study, a coculture system was employed. After 3 days, hAMSCs' secretome was collected and its effects on tumor formation, inflammation, and cell invasion were analyzed using Western blot, scratch assay, qPCR, and ELISA.

**Results:**

The study revealed a decrease in the TGF‐βR/SMADs/c‐MYC signaling pathway and an increase in levels of p15^INK4B^, p21^CIP1^, TNF‐α, and IL‐4/6/8. Additionally, a decrease in invasion was detected in HT‐29 cells treated with hAMSCs.

**Conclusions:**

Our results could be useful in developing a new approach to treating cancer using hAMSCs' secretome.

## Graphical abstract

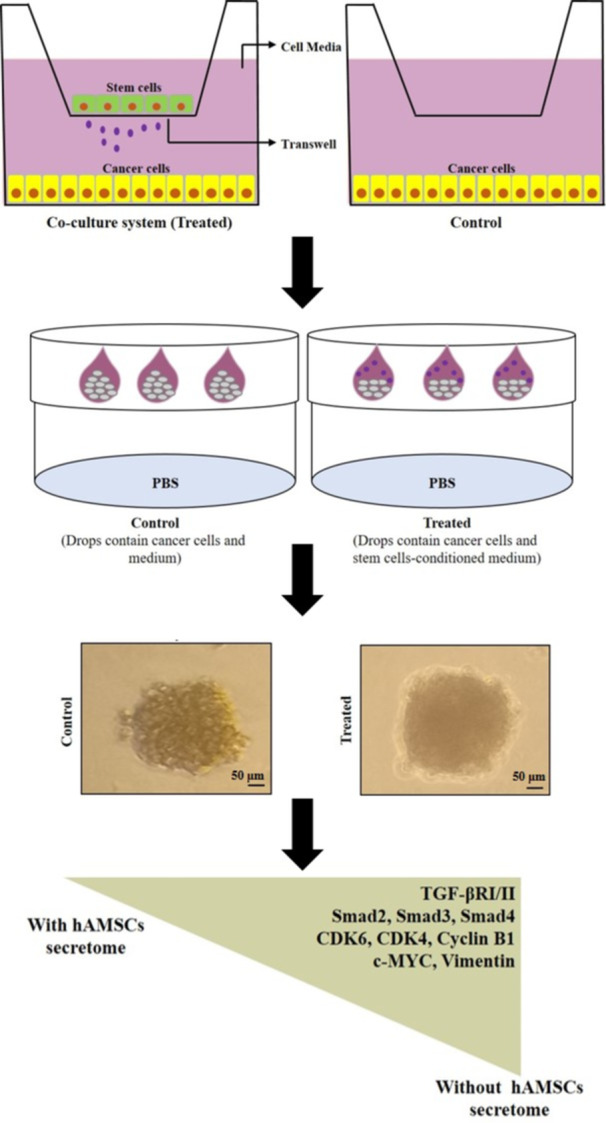

## 1 Introduction

Colon cancer is recognized as one of the deadliest cancers globally. Current cancer treatment methods are not effective; so, a new and potent platform is required. Stem cells are now being thought a valuable and hopeful platform in cancer remedy [1, 2]. Stem cells have special biological characteristics including the ability to secrete bioactive factors, low immunogenicity, and self‐renewal. Among several types of stem cells, mesenchymal stromal cells (MSCs) can be easily separated and grown in vitro from diverse sources, making them a powerful tool in cancer treatment [3, 4]. It should be pointed out that the curative properties of MSCs in colon cancer remedy have been indicated [5].

Tumor necrosis factor alpha (TNF‐α) is an inflammatory‐inducing factor that has been found to promote cancer progression by governing the growth, movement, and invasion of different types of cancer cells [6, 7]. Interleukin‐6 (IL‐6), IL‐8, and IL‐4 also play roles in cancer progression by regulating cellular signal transduction pathways [8, 9, 10].

Transforming growth factor beta (TGF‐β) is a multifunctional cytokine that interacts with its receptor subunits (TGF‐β type I/II) to activate the TGF‐β cascade. Recognized Smad signaling is triggered via the phosphorylation of receptor‐associated Smads, specifically, Smad2 and 3. Once phosphorylated, Smad2 and 3 generate a complex with Smad4, which then moves to the nucleus to connect to promoter regions of TGF‐β target genes [11, 12]. In cancer, TGF‐β has dual roles, acting as a tumor inhibitor in the initial stages of cancer formation and continuing to function as a tumor inhibitor in later stages [13, 14]. Additionally, TGF‐β has been exhibited to have antiproliferative effects by downregulating cell cycle inducers such as the oncogenic c‐MYC and CDK4/6 while simultaneously upregulating CDK inhibiting proteins (CKIs) like p15^INK4B^ and p21^CIP1^ [15, 16].

In this investigation, we attempt to assess the influence of the secretome derived from human amniotic mesenchymal stromal cells (hAMSCs) on the suppressing of migration in HT‐29 colon cancer cells. This will be achieved by examining the TNF‐α/TGF‐βR/c‐MYC signaling pathway and the expression levels of IL‐6/8/4, p15^INK4B^, and p21^CIP^. Through various techniques such as scratch assay, Western blot, ELISA, and qPCR, we evaluated the inhibitory impacts on invasion in HT‐29 cells treated with hAMSCs. Our findings suggest that MSCs may offer a special platform and a new therapeutic approach for the treatment of colon cancer by downregulating TGF‐β receptors I/II, Smad2/3/4, c‐MYC, *Vimentin*, matrix metalloproteinase 9 (MMP9), and CDK4/6 while simultaneously upregulating p15^INK4B^, p21^CIP1^, *E‐cadherin*, TNF‐α, and IL‐6/8/4.

## 2 Materials and Methods

### 2.1 Culture of Cells

hAMSCs were acquired from the Iranian Biological Resource Center. The colon cancer cell line (HT‐29) was sourced from the Pasteur Institute in Tehran, Iran. The cell culture conditions followed the protocols established in our former publication [17].

### 2.2 Cocultivation of HT‐29 and MSCs

Initially, 150,000 HT‐29 cancer cells were plated on the lower face of a six‐well plate. The next day, hAMSCs were introduced at an equal density (hAMSCs: HT‐29 at a ratio of 1:1) on the upper face of a Transwell filter system. The conditions followed the protocols established in our previous publication. After 3 days, the secretome from hAMSCs was harvested and stored at –80°C [17].

### 2.3 Hanging Drop Assay

The hanging drop assay serves as a three‐dimensional cell culture model. In this procedure, HT‐29 cells were grown, trypsinized, and quantified, establishing a concentration of 20 × 10^3^ cells/mL. Drops containing the cells and secretome from hAMSCs were carefully pipetted onto the cap of a 60 mm cultured cell dish. The experiment included both a control group (cells combined with medium) and a sample group (cells combined with hAMSCs secretome). Spheroid formation was observed 3 days post‐seeding [18].

### 2.4 Protein Expression Studies

Protein expression investigations were conducted using various antibodies. The following antibodies were acquired from Santa Cruz Biotechnology: anti‐cyclin B1 (GNS1: sc‐245), anti‐β‐actin (C4: sc‐47778), anti‐c‐MYC (C‐33: sc‐42), anti‐MMP9 (E‐11:sc‐393859), anti‐p21 (F5: sc‐6246), anti‐p15 ^INK4B^ (D12: sc‐271791), anti‐CDK4 (DCS‐35: sc‐23896), anti‐CDK6 (B‐10: sc‐7961), anti‐ TGF‐βRI (D‐1: sc‐518018), anti‐TGF‐βRII (D‐2: sc‐17799), anti‐IL‐8 (C‐11: sc‐376750), and anti‐SMAD3 (38‐Q: sc‐101154). Additionally, anti‐SMAD2 (ab228765), anti‐TNF‐α (ab6671), and anti‐SMAD4 (E‐AB‐34159) were sourced from Abcam and Elabscience, respectively. In brief, HT‐29 cancer cells were collected, lysed, and proteins were delivered to PVDF membrane filters (Millipore). The PVDF membrane was then incubated in solutions with the first antibody (1:300, 90 min at room temperature) and secondary antibody (1:1000, 45 min). All antibodies served as the first antibodies for immunoblotting, following the experimental procedures outlined in our former publication [17]. For stripping, the membrane was placed in the stripping solution (containing mercaptanol, Tris, and SDS) at 37°C for 3 min, then washed with TBST before re‐blocking and re‐probing the membrane.

### 2.5 ELISA

ELISA was utilized to assess the levels of IL‐4 (DY204‐05), IL‐8 (DY208‐05), and IL‐6 (DY206‐05) in the supernatant of HT‐29 cancer cells treated with secretome for 48 h. ELISA kits from R&D Systems, Minneapolis, MN, USA, were used following the manufacturer's instructions. The standard and sample wells were arranged as instructed, then the plates were blocked with film and kept at 37°C for half an hour. Following the rinsing of the plates five times, 50 μL of enzyme‐labeled solution was added to each well and kept at 37°C for half an hour (except for the blank wells). Following another washing step, 50 μL of chromogens A and B were added and kept in the dark at 37°C for a quarter of an hour. The reaction was completed by adding 50 μL of stop agent, and the absorbance was analyzed utilizing a microplate reader (BioRad, iMark) within 15 min at an optical density of 450 nm.

### 2.6 qPCR

After 3 days, drops containing cells were collected, lysed, and then analyzed using qPCR. The qPCR conditions and primer sequences used were previously documented [19]. The experiments were conducted in triplicate.

### 2.7 Scratch Assay

The cells (4 × 10^5^ cells per well) were cultured in six‐well plates for 1 day. A scratch in each well was made utilizing a pipette tip, and then they were kept for an additional 2 days. The migration of cells into the wound was observed and analyzed by measuring the primary wound size and comparing it to the wound size after 2 days using microscopy [19].

### 2.8 Data Analysis

The observations were examined and graphed following our former study [17]. Briefly, SPSS 22.0 (IBM Corp) was used to analyze the data, and GraphPad Prism 7 software was used to create graphs. Additionally, the data were expressed as mean ± standard deviation. Furthermore, the experiments were performed three times, and the groups were compared using an independent samples *t*‐test. Finally, a *p‐*value less than 0.05 was considered statistically significant.

## 3 Results

### 3.1 Induction of TNF‐α in Spheroids

**Figure 1:**
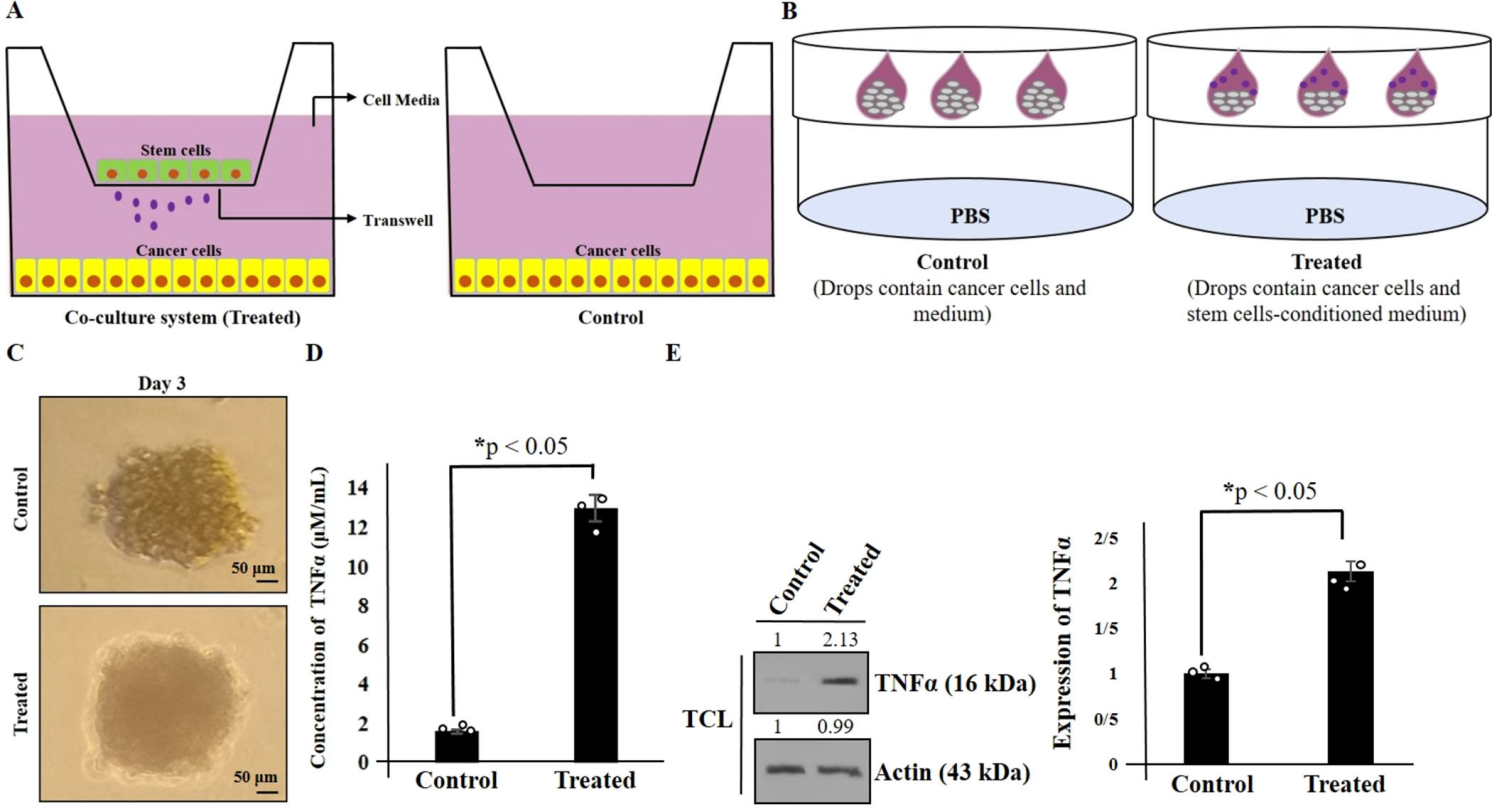
Schematic model of coculture system used in this study (A). Proposed model of 3D cell culture model (hanging drops) was shown (B) (Schematic models of coculture and 3D cell culture were adapted from references [17, 20]). Spheroids formation of HT‐29 colon cancer treated with hAMSCs secretome is shown. Scale bars represent 50 μm (C). The induction of TNF‐α expression of hAMSCs secretome‐treated HT‐29 cells using western blot and ELISA kit in a 3D model (D, E). Actin used as an internal control (TCL: total cell lysate). Data represent mean ± SD of three independent experiments. *p < 0.05 was considered statistically significant.

It was observed that cells can proliferate on all sides within a 3D cell culture model, making it a more accurate representation of in vivo cell actions. Consequently, a hanging drop test was conducted to facilitate the formation of spheroids [18]. In this context, cells were cultured using a Transwell coculture system (Figure 1A). Subsequently, secretome from hAMSCs was collected and used in the hanging drop test to generate spheroids. Consistent with our former study, spheroids were observed after 3 days (Figure 1B,C). Moreover, the involvement of inflammation and inflammatory mediators in promoting colon cancer has been documented [21]. Therefore, the expression of TNF‐α was analyzed using Western blotting and ELISA kits. The cells were cultured with secretome in a 3D model, and after 3 days, spheroids were harvested for analysis via Western blot and ELISA kits. Our results indicated an increase in TNF‐α levels in hAMSCs secretome‐treated HT‐29 cells (Figure 1D,E).

### 3.2 Induction of IL‐6, IL‐8, and IL‐4 in Spheroids

**Figure 2:**
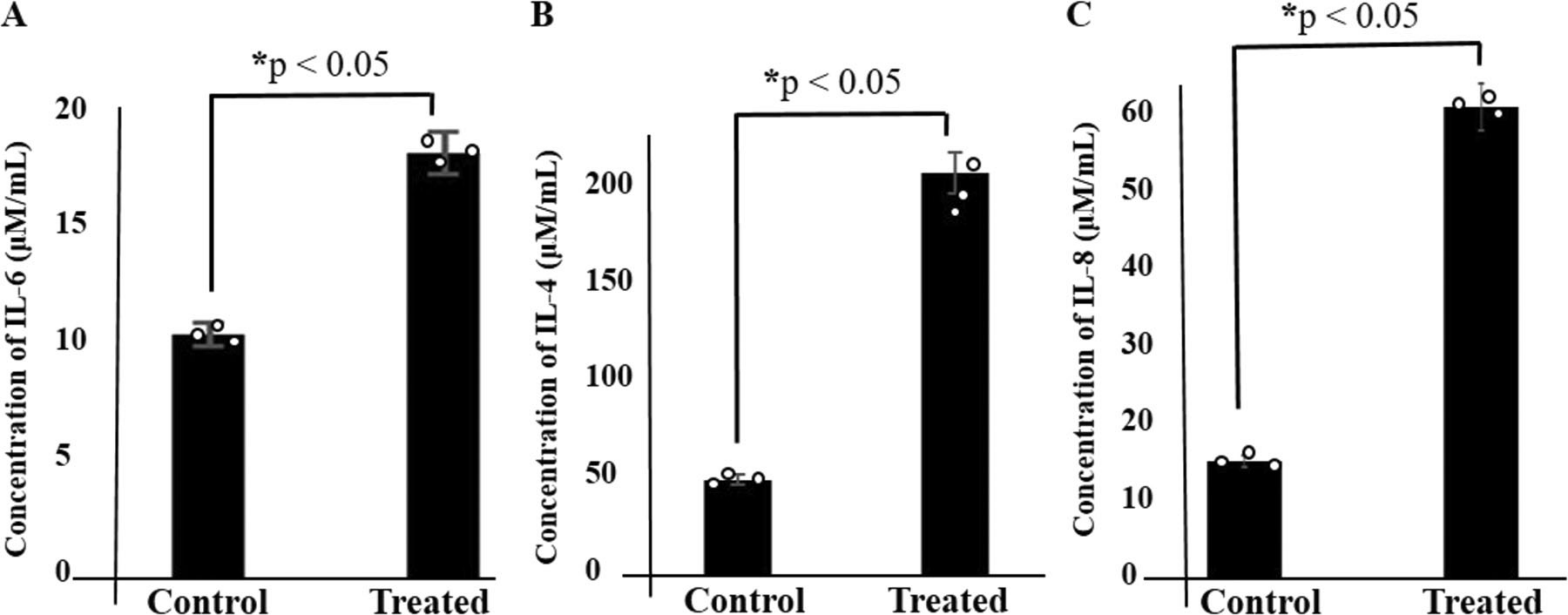
The induction of IL‐6, IL‐4, and IL‐8 expression of hAMSCs secretome‐treated HT‐29 cells using ELISA kit in a 3D model (A–C). Data represent mean ± SD of three independent experiments. *p < 0.05 was considered statistically significant.

The roles of inflammatory response constituents such as IL‐6, IL‐8, and IL‐4 in cancer progression through governing cellular signal transduction pathways have been well‐established [8, 9, 10]. In this study, we measured the expression levels of IL‐6, IL‐8, and IL‐4 utilizing ELISA kits. Initially, the cells were cultured with secretome via a hanging drop technique. After 3 days, the resulting spheroids were harvested and analyzed with ELISA kits. Our findings indicated the induction of IL‐6, IL‐8, and IL‐4 in HT‐29 cells treated with hAMSCs secretome (Figure 2A–C).

### 3.3 Suppression of TGF‐βRI/II, Smad2/Smad3/Smad4, c‐MYC, and Elevation of p15 ^INK4B^ and p21^CIP1^ Expressions in HT‐29 Cells by Secretome

**Figure 3:**
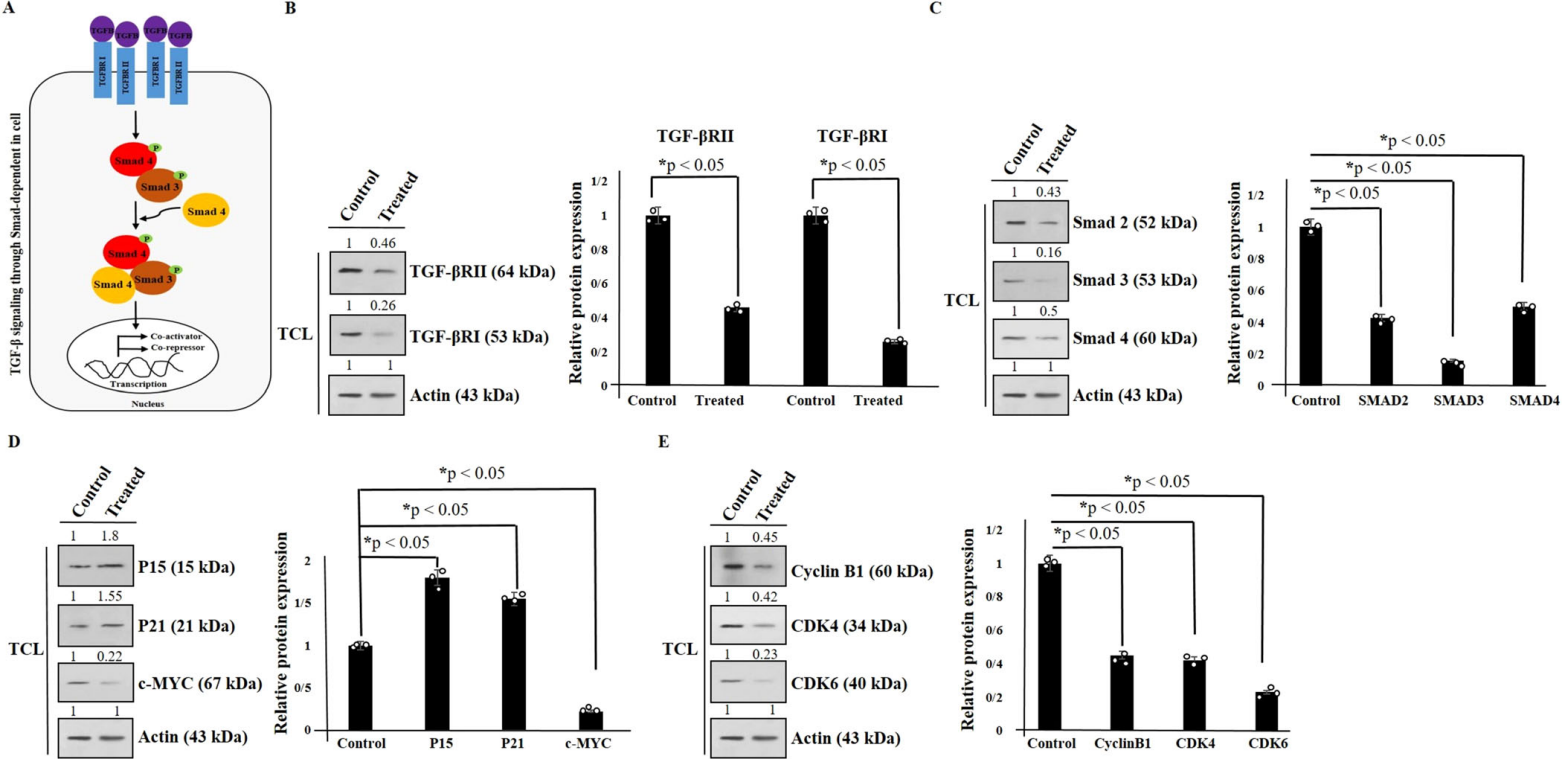
The involvement of c‐MYC/p15 ^INK4B^/p21^CIP1^ in TGF‐β/Smad signaling pathway (A). The expression of TGFβRI/II, Smad2, Smad3, and Smad4 proteins by using western blot in hAMSCs‐treated HT‐29 cells (B, C). The expression of c‐MYC, p15 ^INK4B^, p21^CIP1^, cyclin B1, and CDK4/6 proteins by using western blot in hAMSCs secretome‐treated HT‐29 cells using 3D model is shown (D, E). Actin used as an internal control (TCL: total cell lysate). Data represent mean ± SD of three independent experiments. *p < 0.05 was considered statistically significant.

In the context of the TGF‐β pathway (classical pathway), TGF‐β binds to its receptor subunits (TGF‐βR I/II), activating the TGF‐β signaling cascade (Figure 3A). Therefore, our aim was to assess the influence of hAMSCs secretome on the TGF‐β pathway through Smad‐dependent expressions in HT‐29 cancer cells. The cells were cultured with hAMSCs secretome, and after 3 days of spheroid formation, the spheroids were collected for analysis of TGF‐βR I/II, Smad2, Smad3, and Smad4 protein expression levels (Figure 3B,C). Previous studies have indicated that the activation of Smad protein complexes by TGF‐β leads to the suppression of the transcription factor c‐MYC [22]. Additionally, p15^INK4B^ and p21^CIP1^ have been found to be induced [23, 24, 25]. To assess the expressions of these related proteins, HT‐29 cancer cells were grown with secretome using a 3D model, and after 3 days, spheroids were harvested, lysed, and loaded for western blot assay. As illustrated in Figure 3D,E, we observed an inhibition of c‐MYC and an elevation of p15 ^INK4B^ and p21^CIP1^ expressions in HT‐29 cells treated with hAMSCs secretome. Furthermore, it was determined that p15^INK4B^ can inhibit CDK4/6 [26], prompting us to assess the expression levels of CDK4/6, which revealed a suppression of both CDK4/6 and cyclin B1 expressions (Figure 3E). These findings suggest that the hAMSCs secretome facilitates the downregulation of TGF‐βR I/II, Smad2, Smad3, Smad4, c‐MYC, CDK4/6, and cyclin B1 while simultaneously upregulating p15 ^INK4B^ and p21^CIP1^ in HT‐29 cells.

### 3.4 The Suppression of Epithelial‐Mesenchymal Transition (EMT) in HT‐29 Cancer Cells Treated With hAMSCs via Elevation of E‐Cadherin and Suppression of Vimentin and MMP9

**Figure 4:**
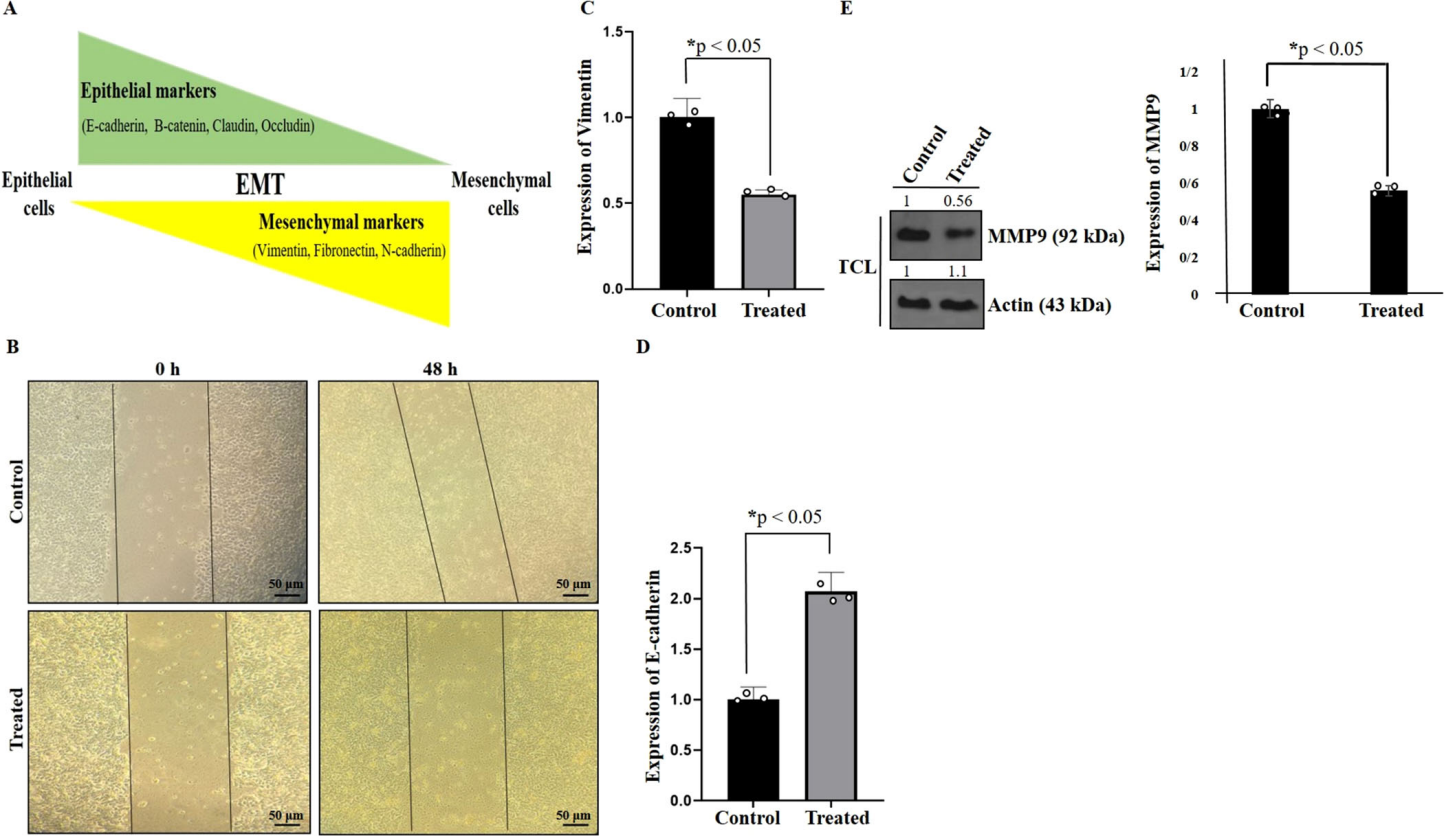
Schematic model of epithelial‐mesenchymal transition (EMT) (A). Effects of hAMSCs on the migration of HT‐29 colon cancer cells in different times (0 and 48 h after treatment). Images were obtained using phase‐contrast microscopy. Scale bars represent 50 μm (B). Relative expression of *E‐cadherin* mRNA and relative expression of *Vimentin* mRNA of HT‐29 colon cancer cells after 72 h treatment with hAMSCs were shown (C, D). Data represent mean ± SD of three independent experiments. * *p* < 0.05 was considered to be statistically significant. Suppression of MMP9 expression in hAMSCs secretome‐treated HT‐29 cells using western blot (E). Actin used as an internal control (TCL: total cell lysate). Data represent mean ± SD of three independent experiments. *p < 0.05 was considered statistically significant.

It has been indicated that TGF‐β receptors can trigger EMT through the Smad signaling pathway [27]. Notably, TGF‐β has been involved in the metastasis of cervical, breast, and ovarian cancer cells [28, 29, 30]. In EMT, epithelial cells lose their cell‐cell junction features and obtain mesenchymal features, leading to increased invasiveness. Concurrently, there is an upsurge in the expression of mesenchymal markers like Vimentin and Snail, while the expression of epithelial markers like E‐cadherin and β‐catenin diminishes (Figure 4A) [31, 32, 33]. To explore the effects of secretome on cell migration, a scratch assay was conducted. The movement of HT‐29 cells was evaluated at the initial time point and after 2 days of treatment with hAMSCs secretome (Figure 4B). Subsequently, RNA was extracted from HT‐29 cells, cDNA synthesis was performed, and qPCR was utilized to evaluate *E‐cadherin* and *Vimentin* expressions (Figure 4C,D). MMP9 expression was also checked using Western blot (Figure 4E). Our observations indicate that the secretome effectively suppressed EMT in HT‐29 cancer cells.

## 4 Discussion

**Figure 5:**
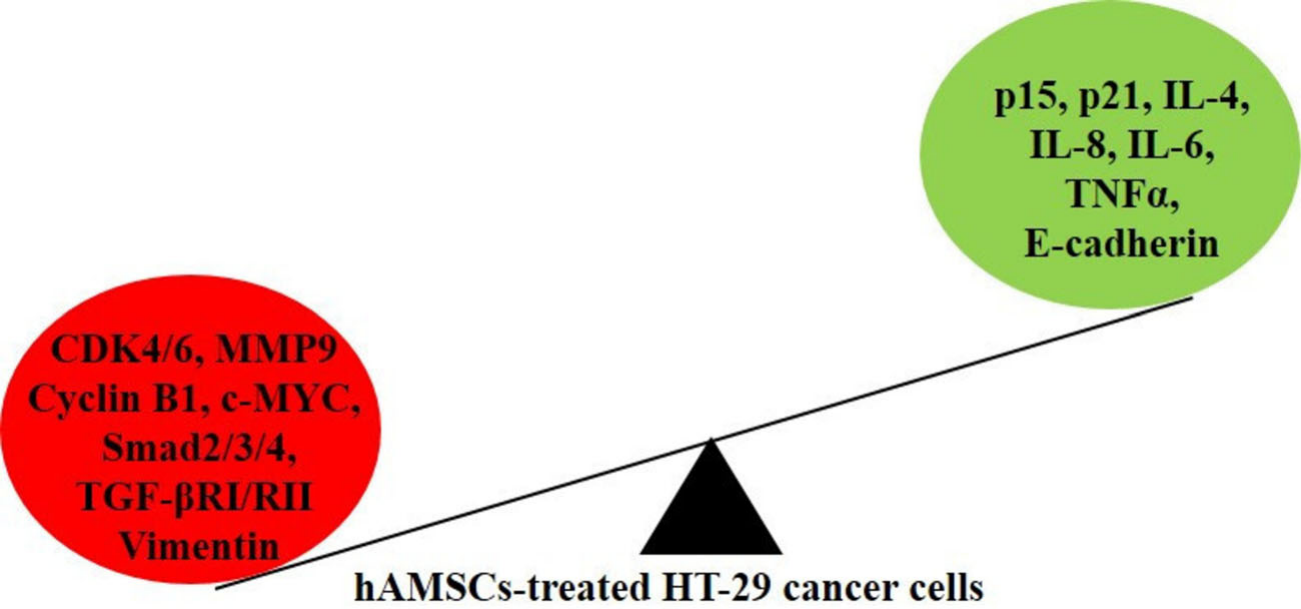
A schematic summary of the present study. hAMSCs secretome enables to induce *E‐cadherin*, p15 ^INK4B^, p21^CIP1^, TNF‐α, IL‐6, IL‐4, and IL‐8 expression and inhibit TGFβRI/II, Smad2, Smad3, Smad4, *Vimentin*, MMP9, and CDK4/6 expressions.

Colon cancer is believed to be one of the main reasons for cancer‐associated mortality globally. Existing cancer treatment approaches have been insufficient, prompting the exploration of innovative and effective therapeutic platforms. This study aims to explore the influences of the secretome from hAMSCs on tumor growth and invasion in HT‐29 cells treated with secretome, utilizing a hanging drop 3D cell culture model. The analysis focuses on the TGF‐β/Smad pathway and major targets, including the transcription factors c‐MYC, p15^INK4B^, and p21^CIP1^. Moreover, IL‐6, IL‐8, IL‐4, and TNF‐α expressions were analyzed. Our findings indicate a downregulation of TGFβRI/II, Smad2, Smad3, and Smad4 in HT‐29 cells treated with hAMSCs secretome. Furthermore, c‐MYC, MMP9, and *Vimentin* were suppressed, while p15^INK4B^ and p21^CIP1^ were upregulated. Importantly, treatment with hAMSCs secretome leads to the induction of *E‐cadherin*, IL‐6, IL‐8, IL‐4, and TNF‐α (Figure 5).

It is noteworthy that IL‐6, IL‐8, IL‐4, TNF‐α, and *TGF‐β* are recognized as protumorigenic factors [34, 35], with elevated levels of TNF‐α and IL‐6 previously documented in colorectal cancer cells (CRC) [21, 36, 37]. Soleimani et al. showed TGF‐β overexpression in CRC progression [38]. Moreover, it was reported that IL‐4 is involved in promoting EMT [39], while IL‐8 is upregulated and induces metastasis and tumor growth in colorectal cancer [40, 41]. Our results imply that the secretome from hAMSCs promotes TNF‐α, IL‐6, IL‐4, and IL‐8 expression while suppressing TGF‐β, SMAD2, SMAD3, and SMAD4 expression in HT‐29 cancer cells.

Numerous pieces of evidence have indicated the overexpression of c‐MYC in many human cancers, including colon carcinomas, where it can be elevated by as much as 40‐fold [42, 43, 44]. Consequently, the ability of hAMSCs' secretome to inhibit c‐MYC expression may represent a significant aspect of its therapeutic potential in colon cancer. Moreover, we observed an increase in p15^INK4B^ and p21^CIP1^ levels in HT‐29 cells treated with hAMSCs. In contrast, a decrease in p15^INK4B^ and p21^CIP1^ levels was noted in pancreatic carcinoma [45]. It should be pointed out that the suppression of c‐MYC and CDK4/6, along with the elevation of CKIs such as p15^INK4B^ and p21^CIP1^, mediates cell cycle arrest in the G1 phase by TGF‐β [15, 16]. However, the dual roles of TGF‐β in tumor cells, the direct effects of the mentioned proteins, their mutual effects, and their interaction with the secretome should be investigated. Therefore, more research is essential to explain the related processes.

Furthermore, our findings indicate that EMT was inhibited in HT‐29 cells treated with hAMSCs, as evidenced by the suppression of *Vimentin* and MMP9 and the elevation of *E‐cadherin*. It is considered that TGF‐β is implicated in EMT via both Smad and non‐Smad signaling pathways [16, 27, 46]. Former research has indicated that hAMSCs' secretome can repress EMT by modulating key target genes, including E‐cadherin, Vimentin, Snail, and Zeb1, as well as the expression of MMP2/MMP9, p38/ERK1/2 phosphorylation, and EGFR/c‐Src expression in different cancer cell types [19, 20, 47, 48]. It appears that hAMSCs' secretome may impede cell migration by suppressing EMT through both Smad and non‐Smad pathways across various cancer cell lines. Moreover, it was indicated that secretome has an inhibitory impact on the migration of cancer cells via suppression of focal adhesion kinase activity and cell adhesion protein expressions in breast cancer cells [49].

The secretome of stem cells possesses many biomolecules, including growth components, chemokines, cytokines, and microRNA (miRNA), which can either stimulate or inhibit tumor growth according to the cellular context [50, 51, 52]. Additionally, the therapeutic effects of secreted TGF‐β1/TGF‐β2 by MSCs have been reported [53]. Our findings collectively indicate that the secretome of hAMSCs can suppress the TGF‐β/Smad/c‐MYC signaling pathway while promoting the expression of p15^INK4B^ and p21^CIP1^, leading to tumor growth inhibition and reduced cell invasion. It is crucial to note that TGF‐β has a dual role in oncogenesis, suggesting that the underlying mechanisms remain unclear and warrant further investigation. As a future study direction, it is suggested to evaluate other components of TGF‐β family, like Inhibins, Activin, and Nodal. Moreover, other molecules (such as miRNAs and various mesenchymal markers) or pathways (such as WNT/β‐catenin, ERK, and JNK) should be analyzed. In this regard, promising target genes through the TGF‐β pathway were discovered [54, 55].

Our study had several limitations: in the tumor microenvironment, not only the direct effects of cytokines but also their mutual interactions should be investigated. Additionally, we analyzed several cytokines (with protumorigenic effects) after treating HT‐29 cancer cells with the secretome. However, more cytokines (with inhibitory effects such as IL‐12, IL‐15, and IL‐18) should be investigated in HT‐29 and other CRC lines. Finally, many potent factors in the TGF‐β pathway (such as SMAD6/7 and FoxO) should be analyzed to better understand the impacts of the secretome on the TGF‐β pathway for designing a hopeful platform in cancer therapy.

## 5 Conclusions

In summary, the findings of this study demonstrate that the secretome of hAMSCs effectively suppresses tumor growth and inhibits the invasion of HT‐29 colon cancer cells via TGF‐β/Smad/c‐MYC/p15^INK4B^ and p21^CIP1^ signaling pathways. Furthermore, we showed the induction of *E‐cadherin*, TNF‐α, IL‐6, IL‐8, and IL‐4 in HT‐29 cells treated with hAMSCs secretome.

## Author Contributions

Fatemeh Safari was responsible for designing the research and conducting the experiments. Both Fatemeh Safari and Mohammad Ali Majdoddin analyzed the data. The paper was written by Fatemeh Safari.

## Ethics Statement

The authors have nothing to report.

## Consent

The authors have nothing to report.

## Conflicts of Interest

The authors declare no conflicts of interest.

## Supporting information

Supplemental fig.

## Data Availability

The information can be acquired upon appropriate demand from the responsible author.
