# Supplementary figures and images for: Investigating the Impact of the Secretome From hAMSCs on HT‐29 Colon Cancer Cells via TNF‐α/TGF‐β/c‐MYC Signaling Pathways Using a Three‐Dimensional Cell Culture Model
Source: Immun Inflamm Dis. 2025 Oct 15;13(10):e70283. doi: 10.1002/iid3.70283 (PMC12521875; doi:10.1002/iid3.70283)

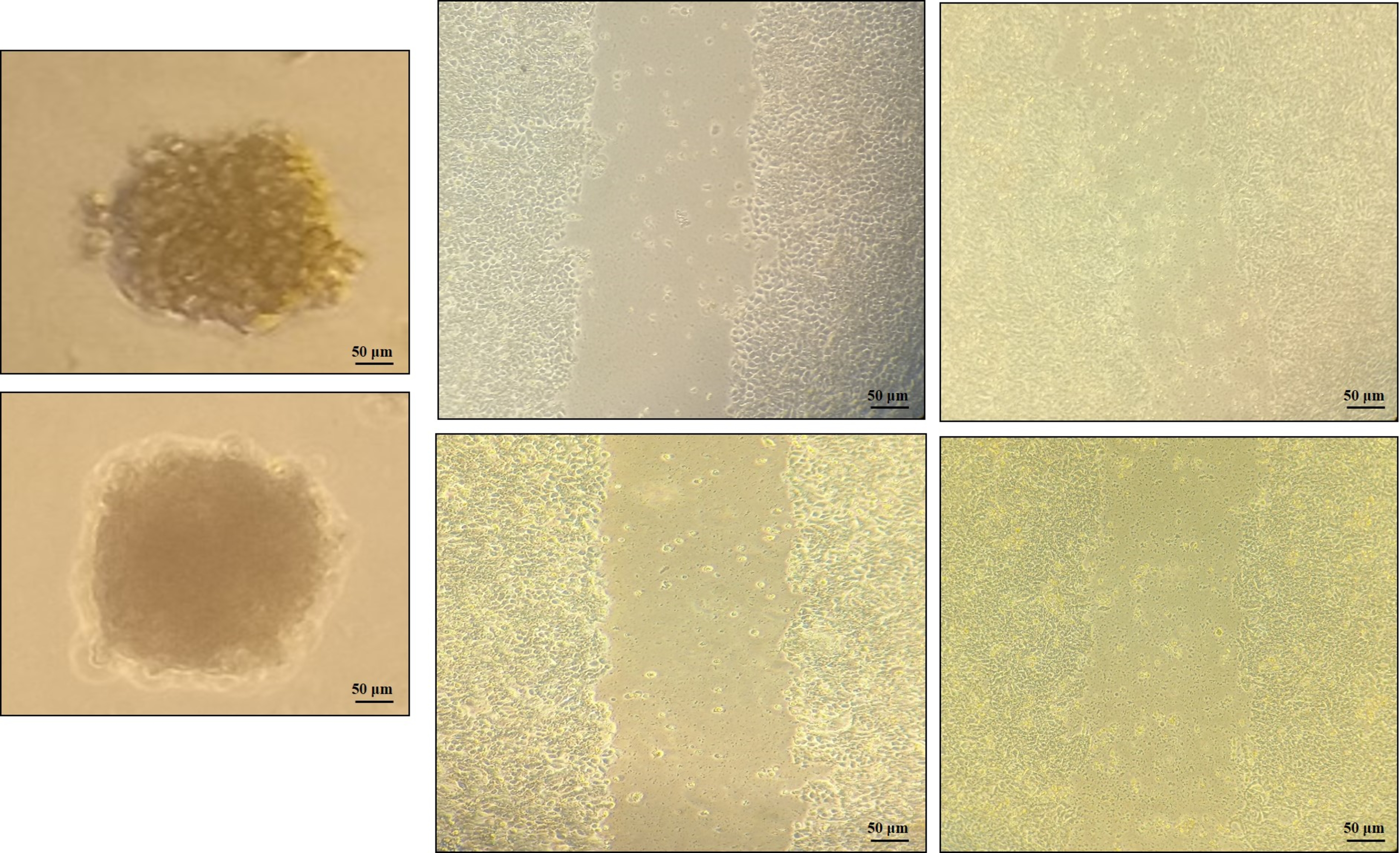

Supplement: Supplementary file 1 — Supplemental fig. [file IID3-13-e70283-s001.jpg]
